# Characterisation of the Ral GTPase inhibitor RBC8 in human and mouse platelets

**DOI:** 10.1016/j.cellsig.2019.03.015

**Published:** 2019-07

**Authors:** Tony G. Walsh, Andreas Wersäll, Alastair W. Poole

**Affiliations:** From the School of Physiology, Pharmacology & Neuroscience, University of Bristol, Bristol BS8 1TD, United Kingdom

**Keywords:** Platelets, Ral GTPase, Secretion, Human, Mouse, CRP, Collagen related peptide, PAR4-AP, Protease activated receptor 4-activating peptide, DKO, Double knockout, WT, Wild-type, PS, Phosphatidylserine, Ral binding protein-1, RalBP1, GST, Glutathione s-transferase, PF4, Platelet factor 4

## Abstract

The Ral GTPases, RalA and RalB, have been implicated in numerous cellular processes, but are most widely known for having regulatory roles in exocytosis. Recently, we demonstrated that deletion of both Ral genes in a platelet-specific mouse gene knockout caused a substantial defect in surface exposure of P-selectin, with only a relatively weak defect in platelet dense granule secretion that did not alter platelet functional responses such as aggregation or thrombus formation. We sought to investigate the function of Rals in human platelets using the recently described Ral inhibitor, RBC8. Initial studies in human platelets confirmed that RBC8 could effectively inhibit Ral GTPase activation, with an IC_50_ of 2.2 μM and 2.3 μM for RalA and RalB, respectively. Functional studies using RBC8 revealed significant, dose-dependent inhibition of platelet aggregation, secretion (α- and dense granule), integrin activation and thrombus formation, while α-granule release of platelet factor 4, Ca^2+^ signalling or phosphatidylserine exposure were unaltered. Subsequent studies in RalAB-null mouse platelets pretreated with RBC8 showed dose-dependent decreases in integrin activation and dense granule secretion, with significant inhibition of platelet aggregation and P-selectin exposure at 10 μM RBC8. This study strongly suggests therefore that although RBC8 is useful as a Ral inhibitor in platelets, it is likely also to have off-target effects in the same concentration range as for Ral inhibition. So, whilst clearly useful as a Ral inhibitor, interpretation of data needs to take this into account when assessing roles for Rals using RBC8.

## Highlights

• RBC8 inhibits RalA/B activation in human platelets.
• Human platelet aggregation, secretion and thrombus formation are inhibited by RBC8.
• Some functional responses in RalAB deficient mouse platelets are inhibited by RBC8.
• In mouse platelets at least, RBC8 elicits off-target effects in a concentration range where RalA/B is also inhibited.

## 1 Introduction

Platelets are essential regulators of haemostasis, limiting blood loss following acute injury, while exacerbated platelet activity in diseased blood vessels is causative of ischaemic tissue damage due to occlusive thrombosis. These events are characterised by dynamic changes in platelet activation responses, including actin cytoskeleton remodelling (shape change and spreading), integrin activation (facilitating platelet aggregation), granule secretion (lysosomes, dense and α-granules), prostaglandin production (thromboxane A_2_) and phosphatidylserine exposure (facilitating thrombin-mediated coagulation) [1,2]. These functional responses are regulated by distinct signalling pathways propagated from various glycoprotein (GP) receptors (GPVI, GPIb-IX-V), G protein-coupled receptors (GPCRs – PAR1/4, TP, P2Y_1/12_) and integrin complexes (α_IIb_β_3_, α_2_β_1_) which converge into common signalling pathways [3]. Among a range of identified signalling molecules important for platelet activation are the small GTPase proteins, in particular Rap1A and Rap1B (Ras family) and Rac1, RhoA, RhoG and Cdc42 (Rho family), which could represent suitable pharmacological targets for antithrombotic therapies [[4], [5], [6]].

Ral GTPases (RalA and RalB) are ubiquitously expressed members of the Ras subfamily of GTPases and are particularly abundant in the brain, testes and platelets [7]. Besides Rap1A and 1B, which have in excess of 100,000 copies/platelet, RalA and RalB are among the more highly expressed Ras GTPase members with 3400 and 6800 copies/platelet, respectively [8,9]. They target numerous effectors including Ral-binding protein 1 (RalBP1), phospholipase D and the exocyst complex to regulate cell polarity, exocytosis, autophagy and various cellular responses associated with tumorigenesis and metastasis [[10], [11], [12], [13], [14]]. In platelets, Rals have been previously shown to be activated in response to various agonists, dependent on rises in cytosolic Ca^2+^ [7]. A further study suggested a role for Rals in regulating platelet dense granule secretion in human platelets, which is consistent with reports in other cell types [15,16]. We recently showed however a restricted role for Rals in regulating exposure of P-selectin on the plasma membrane of mouse platelets [17]. Conditional deletion of either RalA or RalB in platelets showed no substantial alterations in P-selectin exposure, however deletion of both RalA and RalB (double knockout, DKO) in platelets produced a pronounced defect in this response. This confirmed a redundancy between Rals, which had been previously reported [13]. However, the defect was unusual in that release of soluble α-granule content, such as platelet factor 4 (PF4), was largely unaltered. This suggested a specific and novel role for Rals, specifically in the control of P-selectin exposure on the platelet surface.

Importantly, other aspects of platelet function (aggregation, spreading, in vitro and in vivo thrombosis) important for haemostasis and thrombosis were unaltered in Ral DKO platelets and mice. Beyond haemostasis and thrombosis however, platelets have been implicated in numerous pathophysiological processes, in which secreted biomolecules from platelet α-granules are likely to play major roles [18,19]. For instance, platelet-expressed P-selectin is crucial in mediating platelet-leukocyte/immune cell and platelet-endothelial cell interactions involved in inflammatory responses [20]. Therefore, since we have shown them to be critically selective for the regulation of surface expression of P-selectin, platelet Ral GTPases may represent a possible candidate for targeting inflammatory diseases in which platelets have well-established roles in, in particular atherosclerosis and cancer [21,22]. We recently showed that the platelet-specific RalAB DKO mice have delayed onset of clinical signs in an inflammatory bowel disease model [17]. However, despite similarities in relative abundance of both Rals between mouse and human platelets, it is still important to clarify if Rals perform similar roles in human platelets, which could pave the way for future therapeutic approaches targeting platelet P-selectin release [9,23].

The development of selective inhibitors targeting small GTPases has posed significant challenges for researchers. There is substantial interested in targeting these molecules, most notably in the field of oncology where the development of direct Ras inhibitors are often referred to as the “Holy Grail” of cancer therapeutics [24]. Numerous targeting strategies have been sought to overcome this, including the specific targeting of downstream effectors of Ras [25]. Being downstream of Ras, RalA and RalB have gained increasing interest as potential targets in the treatment of cancer, leading to the development of the first commercially available Ral inhibitor, RBC8 [26]. This non-competitive compound binds to an allosteric site on GDP-bound Rals, locking them in an inactive state, which blocks their interaction with the effector protein, Ral binding protein 1 (RalBP1). Importantly, RBC8 showed selectivity over GTPases Ras and Rho, and functionally it could suppress tumor xenograft growth in mice, which is consistent with a previous publication describing redundant roles for RalA and RalB in tumorigenesis [13,26]. Based on our findings in RalAB DKO platelets, we sought to investigate the role of Ral GTPases in human platelets using RBC8 as a molecular tool to block Ral function, while utilising RalAB DKO platelets to assess the specificity of RBC8 in similar functional assays. We confirm that RBC8 effectively reduces RalA and RalB activation in human platelets, but the compound was able to inhibit also functional responses that were not seen to be inhibited by gene deletion in the DKO mouse. Furthermore, RBC8 inhibited various aspects of platelet function in RalAB-null mouse platelets, suggesting RBC8 also has off target activity in platelets, in the concentration range where Ral is inhibited. We conclude therefore that, although clearly useful as a reagent to effectively inhibit RalA and RalB activity, in platelets at least there are likely to be other targets for RBC8. So, whilst clearly useful as a Ral inhibitor, interpretation of data needs to take this into account when assessing roles for Rals in platelets using RBC8.

## 2 Materials and methods

### 2.1 Materials

RBC8 was provided as a generous gift from Prof. Theodorescu (Cedars-Sinai, USA) and reconstituted in DMSO (used as vehicle control). Platelet agonists: Cross-linked collagen related peptide (CRP) was purchased from Prof. Richard Farndale (University of Cambridge, UK), adenosine diphosphate (ADP) and thrombin was from Sigma-Aldrich (Poole, UK) and protease activated receptor activating peptide (PAR4-AP) was from Peptide Synthetics (Hampshire, UK). For western blotting, RalA (# 3526) and RalB (# 3523) antibodies were from Cell Signalling Technology (New England Biolabs, Hitchin, UK) and BD Biosciences (Oxford, UK, # 610222), and the Rap1 antibody (#sc-65) was from Santa Cruz Biotechnology (Insight Biotechnology, Middlesex, UK). Horseradish peroxidase (HRP)-conjugated secondary antibodies were from Jackson Immunoresearch (Stratech Scientific, Glasgow, UK). EDTA-free Protease inhibitors (# 11836170001) were from Roche (West Sussex, UK). For human platelet flow cytometry experiments: FITC-conjugated PAC-1 (# 340507) and PE-conjugated CD62P/P-selectin (# 561921) antibodies from BD Biosciences were used for measuring integrin α_IIb_β_3_ activation and α-granule secretion, respectively. For mouse platelet flow cytometry experiments: PE-conjugated JON/A (# M023-2) and FITC-conjugated CD62P/P-selectin (# M130-1) antibodies from Emfret Analytics (Eibelstadt, Germany) were used for measuring integrin α_IIb_β_3_ activation and α-granule secretion, respectively. For mouse platelet-leukocyte aggregate studies, FITC-conjugated CD41 antibody (# MCA2245F) was from Bio-Rad (Hertfordshire, UK) and PE-conjugated CD45 antibody (# 103101) was supplied by BioLegend (London, UK). DiOC_6_ iodide for labelling whole blood was from Enzo Life Sciences (Exeter, UK, # ENZ-52303). Fura-2 AM (# F1221) to measure intracellular calcium (Ca^2+^) and Alexa Fluor 488-conjugated annexin V (# A13201) to measure phosphatidylserine (PS) exposure, were from Molecular Probes™ (Thermo Fisher Scientific, Loughborough, UK). Human PF4 DuoSet ELISA (# DY795) was purchased from R&D Systems (Abingdon, UK). Unless stated, all other reagents were purchased from Sigma-Aldrich.

### 2.2 Human and mouse platelet isolation

Both human and animal studies were approved by the local research ethics committee at the University of Bristol. For human platelet studies, informed consent was obtained from healthy, drug/anti-platelet free volunteers in accordance with the declaration of Helsinki. Venous blood was drawn into a syringe containing 4% trisodium citrate (1:9 v/v) and platelets were prepared as previously described [27]. For mouse platelet studies, conditional RalAB deficient mice in the megakaryocyte-platelet lineage were generated using transgenic PF4-Cre mice as previously described [17]. RalA^flox/flox^:RalB^flox/flox^ mice that were Cre^−^ are herein referred to as wild-type (WT); RalA^flox/flox^:RalB^flox/flox^ mice that were Cre^+^ are herein referred to as double knockout (DKO). These mice were bred and maintained in accordance with the UK Home Office regulations and Animals (Scientific Procedures) Act of 1986 (PPL No: 300/3445 held by Prof. Alastair Poole). Age- (8–24 weeks) and sex-matched mice were used for all experiments. Mice were euthanized by CO_2_ asphyxiation and blood was drawn from the inferior vena cava into a syringe containing 4% trisodium citrate (1:9 v/v). Washed mouse platelets were prepared as previously described [28]. Both human and mouse platelets were allowed to recover for at least 30 min at 30 °C prior to experimentation. Pre-treatment of whole blood/washed platelets with the Ral inhibitor, RBC8, or vehicle control (0.2% DMSO) was for 15 min prior to agonist addition.

### 2.3 Glutathione S-transferase (GST)-RalBP1 purification

A pGEX4T3-GST-RalBP1 expression vector, containing amino acids 397–518 of human RalBP1, was generated as previously described [7]. The vector was transformed in BL21(DE3)pLysS protease-free bacteria (Agilent Technologies, CA, USA) and grown to log phase in lysogeny broth media before induction with 0.2 mM isopropyl β-D-1-thiogalactopyranoisde (IPTG) for 3 h at 30 °C. Bacteria were pelleted at 6000 rpm for 30 min (4 °C) in a Sorvall RC 6 Plus centrifuge (Thermo Fisher Scientific, SLA-1500 rotor) and lysed in 15 mL of Phosphate-buffered saline (PBS) lysis buffer (1 x PBS, 0.1% Triton X-100, 1 mM dithiothreitol (DTT), protease inhibitor tablet). The bacterial lysate was freeze-thawed, then supplemented with 100 μg/mL lysozyme before sonication, followed by lysate clarification at 12,000 rpm for 30 min (4 °C, Sorvall SS34 rotor). GST-RalBP1 was then purified by incubation with Glutathione Sepharose™ 4B (Thermo Fisher Scientific) for 90 min on a roller, followed by 3× washes in lysis buffer, 1 x wash in high salt buffer (0.5 M NaCl, 0.1% Triton X-100) and resuspension in storage buffer (1 x PBS, 50% glycerol, 1 mM DTT, 0.1% sodium azide).

### 2.4 Pulldown assay and immunoblotting

Following RBC8/vehicle treatment, human platelets at 4 × 10^8^/mL were stimulated, lysed and incubated with the GST-RalBP1 bait protein to measure RalA/B activity and a GST-tagged Rap binding domain of RalGDS (GST-RalGDS-RBD) bait to measure Rap1 activity [17,29]. Platelet lysate (30 μL) was retained from each sample as loading control inputs. Pull-down and input samples were separated by electrophoresis on 12% Tris-Glycine sodium dodecyl sulfate-polyacrylamide gels (SDS-PAGE) and transferred to Immobilon-P membrane (Millipore, Hertfordshire, UK). Membranes were then blocked in 5% bovine serum albumin (BSA – 1 h at RT) before incubation with primary (overnight at 4 °C) and secondary (1 h at RT) antibodies, with several washes in Tris-buffered saline (TBS) containing 0.1% Tween in between. Immunoblots were developed using an enhanced chemiluminescence (ECL) detection system. Densitometry was performed with Image J (Version 1.46, NIH).

### 2.5 Lumi-aggregometry

Simultaneous assessment of platelet aggregation and dense granule secretion (ATP release) was performed using a CHRONO-LOG® 700 lumi-aggregometer (Labmedics, Oxfordshire, UK) as previously described [30].

### 2.6 Flow cytometry assays

To measure integrin activation and α-granule release, washed human/mouse platelets were pre-incubated with 5 μL FITC-PAC1 (human)/PE-JON/A (mouse) and 2.5 μL PE (human)/FITC (mouse)-P-selectin antibodies. Similarly, for phosphatidylserine exposure (PS) analysis, washed human platelets were incubated with 2 μL Alexa Fluor 488-conjugated annexin V. Following RBC8/vehicle pretreatment, samples were then stimulated with indicated concentrations of platelet agonists for 10 min at a final platelet concentration of 2 × 10^7^/mL in the presence of 1 mM CaCl_2_. Samples were analysed on an Accuri™ C6 Plus flow cytometer (BD) with 10,000 gated events/sample.

### 2.7 Ex vivo thrombus formation

Analysis of human whole blood thrombus formation under flowing conditions was performed as previously described [31].

### 2.8 Platelet factor 4 (PF4) ELISA

Secretion of the α-granule protein, PF4, was measured using a commercially available colorimetric ELISA kit. In brief, human washed platelets (2 × 10^8^/mL) pretreated with RBC8/vehicle were recalcified with 1 mM CaCl_2_ immediately prior to stimulation with indicated concentrations of CRP for 10 min at 37 °C. Platelets were then pelleted (500 *g* for 2 min) to isolate releasates, with a further 2 x pulse spins for 30 s to remove any debris. Control total samples were generated by lysing platelets with an equal volume of 1% Triton X-100. Subsequent ELISA steps were performed as per manufacturer's instructions with releasate and total samples diluted 1/5000 and 1/10,000 in reagent diluent (1% BSA in PBS), respectively. Absorbance values were determined using a Tecan Infinite® M200 Pro plate reader (Reading, UK).

### 2.9 Ca^2+^signalling

Human platelet rich plasma (PRP) isolated from whole blood was incubated with 4 μM Fura-2 AM for 45 min at 30 °C in the dark. Dye-loaded washed platelets were allowed to recover and were then pretreated with RBC8/vehicle before been adjusted to 1 × 10^8^/mL. Samples were recalcified with 1 mM CaCl_2_ and analysed immediately for basal Ca^2+^ values on a plate reader (10 cycles on a Tecan Infinite® M200 Pro), before the addition of CRP agonist. Changes in intracellular Ca^2+^ were then monitored for 40 cycles (approximately 7.5 min), with area under the curve values generated for individual Ca^2+^ responses.

### 2.10 Platelet-leukocyte aggregates

Murine platelet-leukocyte aggregate formation was measured by flow cytometry as previously described [17].

### 2.11 Statistical analysis

Statistical analysis was performed using Graph Pad Prism 7 software. All data are representative of a minimum of 3 independent experiments, presented as mean ± standard deviation (s.d.). Statistical differences were determined using one-way and two-way ANOVA with Bonferroni's post hoc test. *P < 0.05 was considered statistically significant.

## 3 Results & discussion

Targeting specific signalling molecules in anucleate human platelets is often hampered by the availability of selective pharmacological inhibitors. As a result, the field of platelet biology is critically reliant on studies using genetically modified mice or blood from patients with inherited bleeding disorders due to mutations in genes regulating haemostasis [32]. Our recent discovery in mouse platelets suggested a critical role for the Ral GTPases, RalA and RalB, in regulating secretion of P-selectin [17]. This finding opens therapeutic avenues for targeting platelet-mediated inflammatory disorders requiring platelet expression of P-selectin, and in our study we showed that platelet-specific deletion of RalA and RalB significantly slowed the onset of symptoms in a mouse model of inflammatory bowel disease. We therefore set out to assess the role of Rals in human platelets using the recently described Ral inhibitor RBC8 [26].

**Fig. 1:**
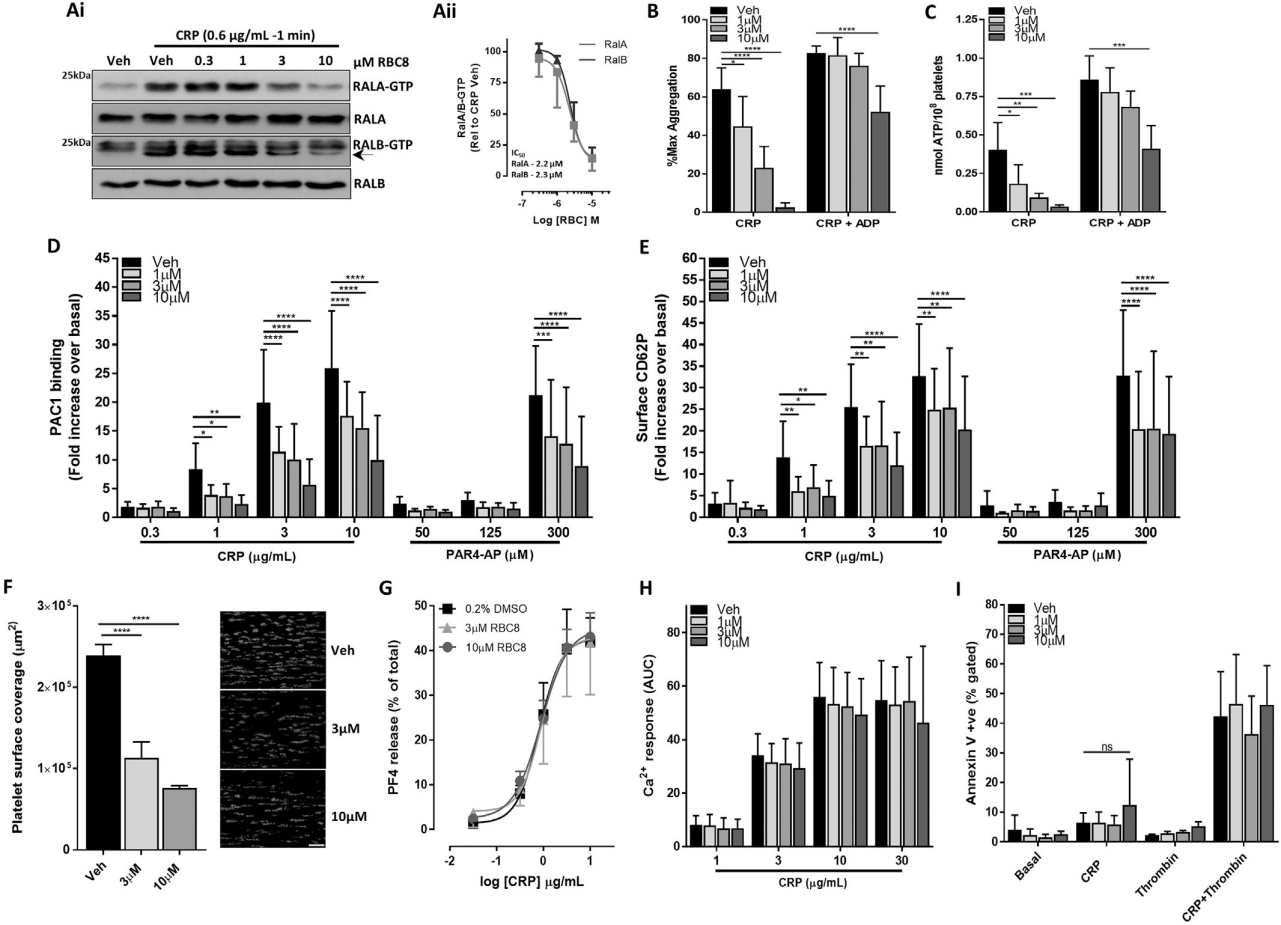
RBC8 inhibits RalA/B activity and specific functional responses in human platelets. (Ai) Washed platelets (4 × 10^8^/mL) pretreated with indicated concentrations of RBC8 were stimulated with CRP and monitored for RalA and RalB-GTP levels using GST-RalBP1 binding protein as bait, with loading controls for total RalA/B content. The arrow is aligned with the RalB-GTP specific signal. (Aii) Densitometric analysis of RalA/B-GTP levels, normalised for total RalA/B loading, were expressed relative to CRP-treated vehicle (0.2% DMSO), with IC_50_ calculation. (B and C) Washed platelets (2 × 10^8^/mL) pretreated with vehicle or RBC8 were assessed for CRP (0.6 μg/mL)-induced aggregation (B) and dense granule secretion of ATP (C), +/− 10 μM ADP, using lumi-aggregometry. (D and E) Following pretreatment with vehicle or RBC8, washed platelets (2 × 10^7^/mL) were activated with indicated concentrations of CRP or PAR4-AP for 10 min and monitored for integrin α_IIb_β_3_ activation (D) and α-granule secretion (E) by PAC1 and P-selectin antibodies, respectively. (F) Anticoagulated whole blood, loaded with 2 μM DiOC6 was pre-treated with vehicle or RBC8 and perfused over collagen-coated surfaces at 1000s^−1^ for 6 min. Total platelet surface coverage (μm^2^) was calculated, with representative confocal images (scale bar = 50 μm). (G) Washed platelets (2 × 10^8^/mL) pretreated with vehicle or RBC8 were stimulated with different CRP concentrations and monitored for release of the α-granule protein, platelet factor 4 (PF4) by ELISA. Secreted PF4 values are expressed relative to total PF4 content from lysed platelets. (H) Washed platelets (1 × 10^8^/mL) in platelet rich plasma (PRP) were loaded with 4 μM Fura-2 AM, pretreated with vehicle or RBC8 (concentration indicated) and stimulated with indicated concentrations of CRP for 7.5 min. Results are displayed as area under the curve (AUC). (I) Changes in phosphatidylserine (PS) exposure were monitored with Annexin V-488 binding in washed platelets (2 × 10^7^/mL) pretreated with RBC and stimulated for 10 min with CRP (5 μg/mL), thrombin (1 U/mL) or combined CRP + thrombin (5 μg/mL + 1 U/mL, respectively) in the presence of 1 mM CaCl_2_. Data are mean ± s.d., n = 3 for (Aii, F, G and I), n = 5 for (B and C), n = 6 for (D and E) and n = 4 for (H); *p < 0.05, **p < 0.01, ***p < 0.001, ****p < 0.0001 vs. indicated sample; (ns) not significant.

Initial experiments confirmed that RBC8 effectively inhibited both RalA and RalB activation in an identical, dose-dependent manner following platelet stimulation with the GPVI-specific ligand, CRP (Fig. 1Ai). Non-specific, upper bands were observed when immunoblotting for activated RalB, with the specific ‘GTP’ signal denoted by the arrow (Fig. 1 Ai). The half-maximum inhibitory value (IC_50_) of RBC8 for RalA and RalB was 2.2 μM and 2.3 μM, respectively (Fig. 1 Aii), which is relatively similar to reported IC_50_ values of 3.5 and 3.4 μM in H2122 and H358 cells, respectively [26]. Having confirmed the inhibitory effect of RBC8 on Ral activity, subsequent experiments set out to assess the effects of RBC8 treatment on platelet functional responses. We specifically chose a threshold concentration of CRP (0.6 μg/mL) as we had previously observed a relatively weak, but statistically significant reduction in dense granule secretion (ATP release), but not aggregation, using this concentration in Ral DKO mouse platelets [17]. With this, we observed a dose-dependent inhibitory effect of RBC8 on human platelet aggregation (Fig. 1B), with a concomitant decrease in dense granule secretion (Fig. 1C). Secretion of ADP from platelet dense granules is an important autocrine/paracrine signalling mediator of GPVI platelet responses and we therefore used ADP “rescue” experiments with exogenously added ADP (10 μM) to understand the mechanism through which RBC8 inhibits human platelet aggregation [33,34]. Notably, exogenous ADP fully recovered the aggregation defect in the 1 and 3 μM RBC8-treated platelet samples, but not completely in 10 μM RBC8 samples (Fig. 1B). This suggests that RBC8, particularly within the IC_50_ range (1–3 μM) reduces ADP secretion necessary for full aggregation responses, but at the 10 μM dose there is an ADP-independent component to RBC8-mediated reduction in platelet aggregation.

Previously, we established that genetic deletion of Rals in mouse platelets causes a substantial reduction in P-selectin surface exposure, without a significant change in integrin α_IIb_β_3_ activation [17]. Using the same flow cytometry assays, we investigated the effect of RBC8 on human platelet responses. Here, RBC8 significantly decreased both readouts of activation in human platelets and these reductions were not agonist-dependent as significant decreases were observed in response to both CRP and PAR4-AP (Fig. 1D and E). While the reduction in P-selectin exposure with RBC8 is consistent with responses in mouse Ral DKO platelets, the decrease in integrin activation with RBC8 was a noticeable divergence in functional responses between RBC8-treated human platelets and mouse Ral DKO platelets. Similarly, we observed a pronounced defect in thrombus formation in vitro in RBC8-treated whole human blood perfused over a collagen-coated surface; a defect which was not apparent in whole blood from Ral DKO mice (Fig. 1F). However, considering the effect of RBC8 on human platelet aggregation, granule secretion and integrin activation, it was not entirely unexpected to observe defective platelet thrombus formation in RBC8-treated whole blood [35]. This finding does also support the efficacy of using RBC8 in native environments such as whole blood, consistent with the seminal paper by Yan et al. reporting decreases in tumor growth from in vivo studies [26].

Further experiments in RBC8-treated human platelets assessed the soluble release of the α-granule marker, PF4, Ca^2+^ mobilisation and phosphatidylserine (PS) exposure (Fig. 1G–I). Here, platelet responses following RBC8 treatment generally showed no effect compared with vehicle/DMSO-treated platelets. The lack of defect in PF4 secretion with RBC8 treatment is important, and aligned with our observations in Ral DKO mouse platelets that show a major defect in P selectin expression with no defect in PF4 release [17]. Furthermore, the absence of altered Ca^2+^ signalling with RBC8 is consistent with previous reports demonstrating that Ral activity is downstream of Ca^2+^ signalling, as an increase in cytosolic Ca^2+^, either due to release from intracellular stores and/or cellular influx, is essential for Ral activation [7]. These rises in cytosolic Ca^2+^ are also important for platelet procoagulant function, as measured by annexin V binding to exposed PS, and therefore the absence of altered PS responses with RBC8 is also unsurprising [36]. Importantly, RBC8 did not alter basal/unstimulated annexin V binding values in unstimulated platelets, confirming that the compound (between 1 and 10 μM) does not non-specifically induce apoptosis in resting platelets (Fig. 1I).

**Fig. 2:**
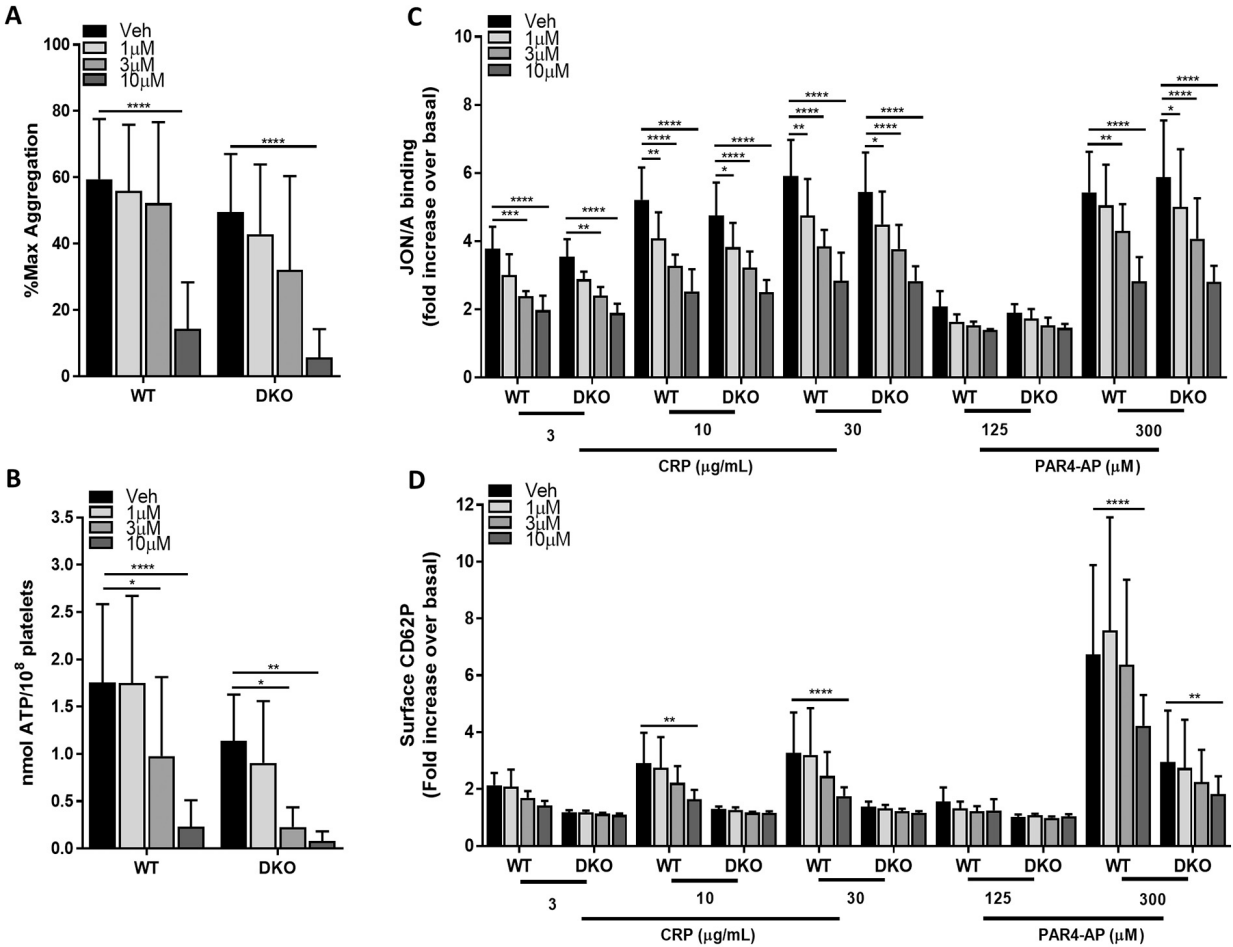
Concentration-dependent inhibition of platelet function by RBC8 in murine RalAB double knockout (DKO) platelets. (A and B) Washed platelets (2 × 10^8^/mL) from WT and DKO mice were pretreated with indicated concentrations of RBC8 or vehicle (0.2% DMSO) and assessed for platelet aggregation (A) and dense granule secretion (B) by lumi-aggregometry using a threshold concentration of CRP (0.6 μg/mL). (C and D) Following pretreatment with vehicle or RBC8, washed platelets (2 × 10^7^/mL) were stimulated with indicated concentrations of CRP or PAR-4 for 10 min and monitored for integrin α_IIb_β_3_ activation (C) and α-granule secretion of P-selectin (D) by flow cytometry using a JON/A and P-selectin antibody, respectively. Data are mean ± s.d., n = 5, *p < 0.05, **p < 0.01, ***p < 0.001, ****p < 0.0001 vs. indicated sample.

Our observations with RBC8 in human platelets suggested a more wide-ranging role for Rals in platelet function compared to our observations in Ral deficient mouse platelets. Using lumi-aggregometry, 10 μM RBC8 significantly reduced platelet aggregation and ATP secretion responses in both WT and DKO platelets using the threshold concentration of CRP (0.6 μg/mL), while 3–10 μM RBC8 also significantly reduced ATP release (Fig. 2A and B). Further investigations using FACS analysis to assess integrin activation (Fig. 2C) revealed almost identical, dose-dependent reductions in both WT and Ral DKO platelets with RBC8 treatment using either CRP or PAR4-AP as agonist. Here, inhibitory responses with RBC8 were more sensitive to lower (1 μM) concentrations of compound compared to the aggregation/dense granule secretion assay. The reduction in CRP-mediated P-selectin exposure in WT platelets with RBC8 appeared to be dose-dependent, but 10 μM RBC8 was required to significantly suppress P-selectin to levels observed in Ral DKO platelets in the absence (or presence) of RBC8 (Fig. 2D). Furthermore, RBC8 could significantly suppress WT platelet-leukocyte aggregation formation, an effect principally mediated by platelet P-selectin interaction with PSGL-1 on leukocytes (Supplementary Fig. 1) [37]. We had previously demonstrated that Ral DKO platelets have a near complete ablation of CRP-mediated platelet-leukocyte interaction, making it challenging to determine off-target effects of RBC8 with this assay [17]. Using PAR4-AP as agonist, RBC8 appeared less potent at reducing P-selectin levels in WT platelets, although 10 μM RBC8 did significantly decrease the response. However, 10 μM RBC8 did also significantly suppress PAR4-AP-mediated P-selectin exposure in Ral DKO platelets (Fig. 2D).

Overall, our data show that RBC8 elicits off-target effects in mouse platelets as evidenced by numerous inhibitory effects in Ral DKO platelets (Fig. 2A-D). It is therefore possible that similar off-target effects exist for RBC8 in human platelets, however we cannot definitively say that this is the case since the differences in our data may just reflect fundamental differences in Ral function between human and mouse platelets. For instance, Rals may have a more critical role in regulating human platelet dense secretion, as reported by Kawato et al., whereas our observations in Ral deficient mouse platelets suggest a very weak role for Rals in dense granule release, which did not alter platelet aggregation or integrin activation responses [16,17]. If such a difference between species were true, it would help explain why inhibition of human Rals (with RBC8) have a more profound effect on human platelet activation responses, which are critically reliant on secreted ADP amplification signals. Also, compensatory upregulation of specific signalling pathways have been previously reported in transgenic mice and therefore it cannot be excluded that similar issues are present in Ral DKO transgenic mice that could potentially mask Ral specific functions in platelets [38]. However, even at 1 μM RBC8, which has weak inhibitory effects on Ral activation (Fig. 1Aii, approximately 10% inhibition), we observed significant effects of the compound on CRP- and/or PAR4-AP-induced human platelet integrin activation and P-selectin exposure (Fig. 1D and E).

While our experiments suggest that RBC8 is targeting signalling component(s) other than Rals in mouse platelets, it is not clear what those target(s) are likely to be. In the Yan paper which identified RBC8 as a Ral inhibitor, the compound showed no off-target activity towards Ras or RhoA, both of which are activated in response to platelet stimulation [39,40]. The GTPase Rac1 has been shown to be important specifically for GPVI-mediated platelet responses, but Rac1 deficient platelets have defective Ca^2+^ mobilisation and RBC8 did not alter CRP-mediated Ca^2+^ signalling responses (Fig. 1H) [41]. Our observations suggest the target(s) is likely to be a Ca^2+^ sensitive component of platelet signalling pathways that is critical for integrin activation and dense granule secretion, the latter being reinforced by our observations that exogenous ADP could largely recover the platelet aggregation defects with RBC8 treatment (Fig. 1B). Based on this, we suspected the Rap1 isoforms, Rap1a and Rap1b, as likely candidates. Like Rals, they are members of the Ras family of GTPases and are specifically regulated by the calcium (and DAG) sensitive guanine nucleotide exchange factor (GEF), CalDAG GEF1, and are critical regulators of integrin activation and platelet secretory responses [6,42]. However, we did not observe any inhibitory effect of RBC8 (between 1 and 10 μM) on CRP-induced Rap1 activation suggesting the off-target effects are not mediated by Rap1 (Supplementary Fig. 2). We are therefore currently uncertain of the Ral-independent mechanism of RBC8 in platelets.

## 4 Conclusion

The development/discovery of compounds targeting small GTPases is challenging [43,44]. Our data point to RBC8 being efficient and potent as a Ral inhibitor in human and mouse platelets, but that it exhibits some activity beyond just Rals, particularly in mouse platelets. It is however possible that species differences in Ral function and structure could partly explain our observations in human platelets, in which wider functions for Rals may be present than in mouse platelets. For functional assessment of Rals in tissues it is advisable therefore to use a combination of genetic and pharmacological approaches and to be aware of possible species differences.

## Conflict of interest

The authors have no conflicts of interest.

## Contributors

A. Wersäll designed and performed experiments, interpreted results and revised the manuscript. T.G. Walsh designed and performed experiments, interpreted results and wrote the manuscript. A.W. Poole designed research, interpreted results and revised the manuscript.

## Appendix A Supplementary data

Supplementary material Image 1
