# Supplementary material for: Characterisation of the Ral GTPase inhibitor RBC8 in human and mouse platelets
Source: Cell Signal. 2019 Jul;59:34–40. doi: 10.1016/j.cellsig.2019.03.015 (PMC6510928; doi:10.1016/j.cellsig.2019.03.015)
Supplement: Supplementary file 1 — Supplementary material [file mmc1.docx]

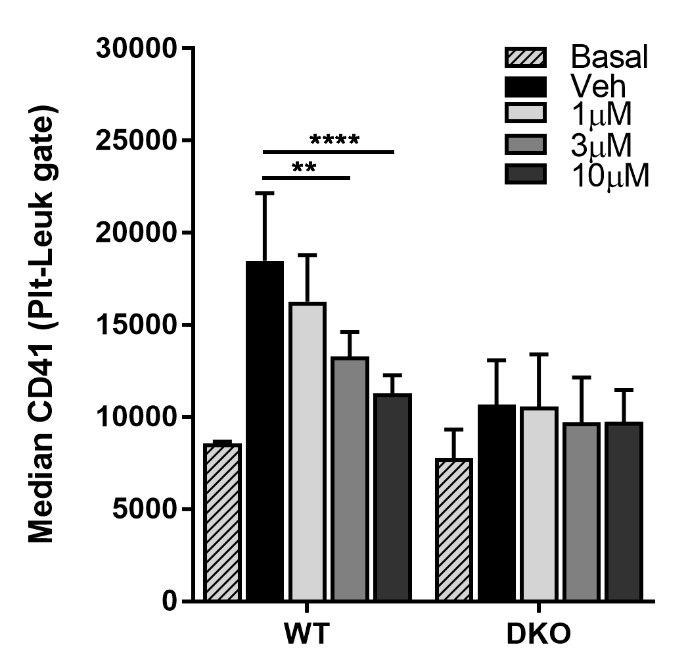


**Supplementary Fig. S1. RBC8 inhibits CRP-dependent platelet-leukocyte aggregation.** Anti-coagulated whole blood from WT and RalAB DKO mice was subjected to red blood cell lysis before samples were left untreated (basal) or stimulated for 10 min with 10 µg/mL CRP following vehicle or RBC pretreatment. Samples were subsequently stained with FITC-conjugated anti-CD41 and PE-conjugated anti-CD45 antibodies for 5 min before quenching with HEPES-Tyrodes. Platelet-leukocyte aggregates were determined by assessing median FITC fluorescence within the gated leukocyte region (Plt-Leuk gate). Data are mean ± s.d, n=3, **p<0.01, ****p<0.0001, vs. indicated sample.


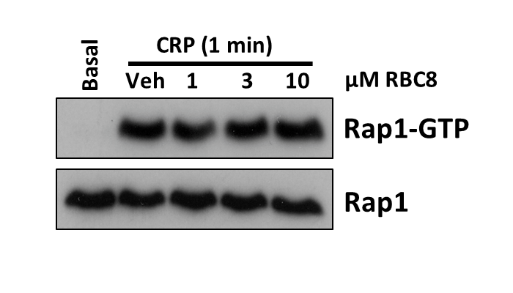


**Supplementary Fig. S2. RBC8 does not target Rap1 activity.** Washed platelets (4 x 10^8^/mL) pretreated with indicated concentrations of RBC8 were stimulated with 0.6 µg/mL CRP for 1 min and assessed for changes in Rap1-GTP levels using the GST-RalGDS-RBD bait protein, with inputs monitored for total Rap1 content. Blot shown is representative of 3 independent experiments.
